# Does breaking up prolonged sitting improve cognitive functions in sedentary adults? A mapping review and hypothesis formulation on the potential physiological mechanisms

**DOI:** 10.1186/s12891-021-04136-5

**Published:** 2021-03-12

**Authors:** Baskaran Chandrasekaran, Arto J. Pesola, Chythra R. Rao, Ashokan Arumugam

**Affiliations:** 1 Department of Exercise and Sport Sciences, Manipal College of Health Professions, Manipal Academy of Higher Education, Manipal, Karnataka India; 2 Active Life Lab, South-Eastern Finland University of Applied Sciences, Mikkeli, Finland; 3 Department of Community Medicine, Kasturba Medical College, Manipal Academy of Higher Education, Manipal, Karnataka India; 4 Department of Physiotherapy, College of Health Sciences, University of Sharjah, P.O.Box 27272, Sharjah, United Arab Emirates; 5 Sustainable Engineering Asset Management Research Group, RISE-Research Institute of Sciences and Engineering, University of Sharjah, P.O.Box: 27272, Sharjah, United Arab Emirates

**Keywords:** Sitting, Workplace, Cognitive function, Physiology, Executive functions, Brain health

## Abstract

**Background:**

Prolonged (excessive) sitting is detrimentally associated with cardiovascular, metabolic and mental health. Moreover, prolonged sitting has been associated with poor executive function, memory, attention and visuospatial skills, which are important cognitive aspects of work performance. Breaking up prolonged sitting with standing or light-intensity exercises at the workplace is recognized as a potential measure in improving cognition. However, preliminary evidence, primarily from acute laboratory experiments, has enabled formulating hypothesis on the possible mechanistic pathways. Hence, the aim of this mapping review is to gather preliminary evidence and substantiate possible physiological mechanisms underpinning the putative effects of breaking prolonged sitting on improving cognitive function among sedentary office workers.

**Mapping method:**

We searched four databases to identify relevant studies that explored the effects of uninterrupted sitting on cognitive function. First, we introduce how prolonged sitting increases the risks of hyperglycemia, autonomic stability, inflammation, adverse hormonal changes and restrictions in cerebral blood flow (CBF) and alters cognitive function. Second, we elucidate the direct and indirect effects of breaking up prolonged sitting time that may prevent a decline in cognitive performance by influencing glycaemic variability, autonomic stability, hormones (brain derived neurotrophic factor, dopamine, serotonin), vascular functions, and CBF. We highlight the importance of breaking up prolonged sitting on metabolic, vascular and endocrine functions, which in turn may improve cognitive functions and eventually foster work productivity. Improved synaptic transmission or neuroplasticity due to increased brain glucose and mitochondrial metabolism, increased endothelial shear and CBF, increased brain neurotrophic factors (dopamine) and accelerated anti-inflammatory functions are some of the hypothetical mechanisms underpinning improved cognitive functions.

**Conclusion:**

We postulate that improving cognitive function by breaking up prolonged sitting periods is biologically plausible with the myriad of (suggested) physiological mechanisms. Future experimental studies to ascertain the aforementioned hypothetical mechanisms and clinical trials to break sedentary behavior and improve cognitive functions in sedentary office workers are warranted.

**Supplementary Information:**

The online version contains supplementary material available at 10.1186/s12891-021-04136-5.

## Background

Due to the rise in computerized jobs, physical labor has reduced significantly in modern workplaces. Sedentary behavior, any awakened behavior characterized by the energy expenditure of fewer than 1.5 METs while in lying, sitting or reclining positions, is conventionally observed in modern workplaces [1]. People employed in office-based work spend a majority of their work time in a seated posture (70–85%), which contributes to their whole day sitting exposure [2, 3]. Desk-based office workers are also found to spend their leisure time in sedentary positions, apart from working hours, compared to their less sedentary counterparts [4]. Considering the fact that office workers spend two-thirds of their wake time in offices, such occupational settings are targeted for promoting physical activity and sedentary behavior interventions [5]. Further, occupational sedentary time is found to be associated with sickness absenteeism and work productivity which may be mitigated through promoting sedentary behavior interventions [6].

Prolonged daily sitting time is adversely associated with all-cause and cardiovascular mortality, and type 2 diabetes incidence [7]. The incidence of cancer and mortality due to excessive sitting time has also recently been established [7]. Amongst other ill effects of excessive sitting, accumulating sitting time in prolonged, uninterrupted bouts can be particularly harmful owing to an increase in cardiometabolic risk biomarkers, type 2 diabetes risk and all-cause mortality [8–11]. Therefore, reducing and breaking up prolonged sitting time is speculated to be a solution to reverse the ill effects associated with the occupational sedentary behavior.

Despite these health outcomes being important from a long-term public health perspective, some more acute work-related outcomes may be relevant in the occupational context. Specifically, there is some experimental evidence on the potential benefits (though partly inconsistent findings) of breaking up prolonged sitting time on fatigue, perceived energy level, cognitive outcomes (executive function, attention and memory), work productivity and workers performance in working-age populations [12–17]. Hence, stakeholders and policy makers may identify evidence linked not only to health-related outcomes, but also to those that are related to work productivity and workers performance.

### Rationale and hypothesis formulation

Though substantial evidence exists to claim the benefits of breaking up prolonged sitting interventions on cardiometabolic risk [18, 19], the physiological mechanisms underpinning the effects of breaking prolonged sitting on cognition in context specific settings remains uncertain. A recent systematic review by Magnon et al. (2020) [20] found no evidence of improved cognition following interventions that intended to decrease sedentary behavior at work. Based on mostly observational evidence, sedentary behavior is linked with many cardiometabolic risks and mechanisms that are associated with altered cognitive functions [20]. Understanding the mechanistic link between sedentary behavior and cognitive function is important in order to design interventions [21] that have the potential to improve cognition and foster work productivity. Wheeler et al. (2016) has explained the plausible mechanistic link between sedentary behavior and cognitive decline based on the glucose-centric view. However, the role of other potential mechanisms such as insulin resistance, low grade inflammation, neuroendocrine dysfunction and altered cerebral blood flow associated with excessive sitting that might influence cognitive decline in adults need to be explored [21].

Though World Health Organisation (WHO) 2020 guidelines reiterated promoting physical activity and sedentary behavior interventions for maintaining adequate mental health and cognition, the direct and indirect effects of occupational sedentary behavior and related interventions on mental health need to be explored further [22]. Though moderate evidence exists for physical activity or sedentary behavior interventions for preventing cognitive decline in elderly, whether similar findings are warranted in desk-based office workers remains uncertain. Further, several research gaps were identified for adults (e.g., the shape of the dose-response curve, the combined associations of physical activity and sedentary behavior with cognition, and the benefits of breaking up sedentary time with light-intensity activity) [23]. Moreover, the optimal balance between occupational activity and sedentary behavior over the course of the workday has not been established [23]. Hence, our mapping review concords with the existing guidelines in establishing the putative physiological mechanisms underpinning sedentary behavior and associated interventions on the cognitive performance in sedentary adults.

## Methods

The present mapping review is framed as advocated by Grant and Booth [24] and presents concepts and hypotheses based on theories stemming from reviews published previously [25, 26]. We used a pragmatic approach with iterative search, a critical interpretive synthesis and a causal mapping method similar to the mapping review of Lorenc et al. (2012) [25]. A criticial interpretive sythesis was required as the main objective of the review was to develop hypothesis based on theories grounded in the studies included in the review [27]. To map out the literature, generate hypotheses and address evidence gap [27], we included review articles and primary studies for understanding the potential physiological mechanisms underpinning the putative effects of breaking up sitting on improving cognitive function. As our goal is to concord with existing evidence and theory, develop concepts and hypotheses, and also identify the need for primary studies investigating the relationship between breaking prolonged sitting and cognitive function, a mapping review was identified to be the most appropriate method.

Four databases (PubMed, Cumulative Index to Nursing and Allied Health Literature (CINAHL), Ovid Medline, Embase) were searched from their inception to December 12, 2020 to identify primary studies and reviews, peer-reviewed and written in English, which measured physical activity or sedentary behavior either subjectively or objectively. The search terms such as “sedentary behavio?r”, “sitting”, “prolonged sitting”, “uninterrupted sitting”, “movement breaks, “microbreaks”, “interrupted sitting”, “replacing or reallocating sitting”, “workplace sitting”, “occupational sitting”, “office sedentary behavior?r”, “cognitive performance”, “cogniti*”, “cognitive function”, “memory”, “executive function*”, “reaction times”, “accuracy”, “attention”, “cognitive flexibility”, “cognitive inhibition”, “information processing speed”, “brain metabolism” were adapted specifically for each database searched. A sample search strategy is provided as an additional file 1.

We searched relevant literature and mapped the physiological mechanisms underpinning the effects of breaking up prolonged sitting on cognition in three steps. First, we introduce how prolonged sitting increases the risks of hyperglycemia, autonomic instability, inflammation, adverse hormonal changes and restrictions in cerebral blood flow (CBF) that might alter the individual’s cognitive function. Second, we elucidate the direct and indirect effects of breaking up sitting on reversing the negative effects associated with prolonged sitting. Third, we suggest a premise that breaking up prolonged sitting time may prevent a decline in cognitive performance by influencing glycemic variability, autonomic stability, hormones (brain derived neurotrophic factor, dopamine, serotonin), vascular functions, and CBF.

We found a substantial amount of evidence investigating the physiological effects of sedentary behavior (excessive sitting) and physical activity on cognitive function. We presented the existing literature in the logical sequence and iterative approach for the plausible physiological mechanisms governing the prolonged sitting and its interruptions on cognitive performance. The iterative steps followed in the present mapping review are provided in a flowchart (additional file: 2).

**Fig. 1:**
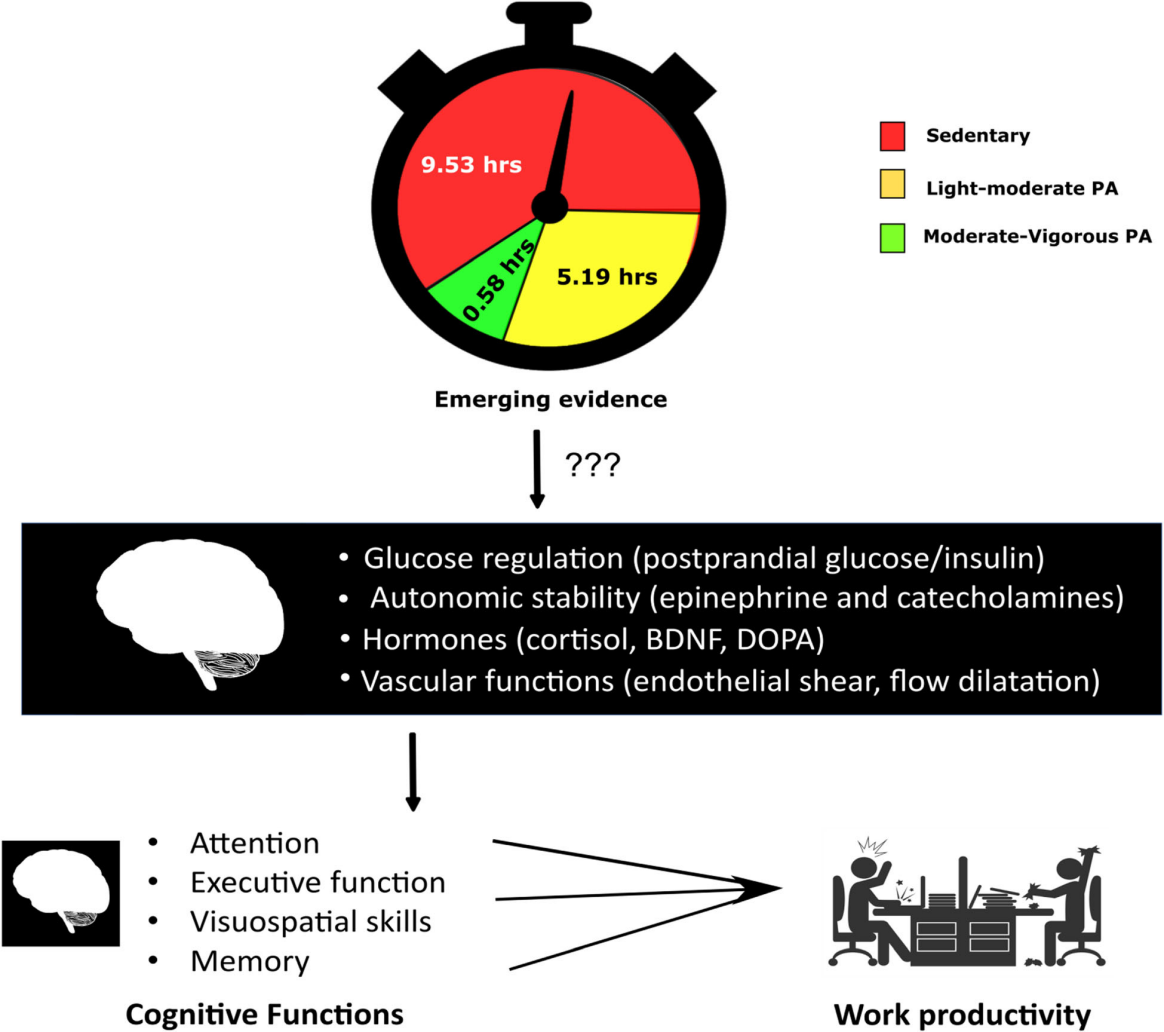
The hypothetical framework of the present mapping review. Substantial evidence is available to support that moderate to vigorous physical activity (MVPA) influences cognitive functions. Evidence is emerging to substantiate the effects of reducing sedentary behavior and breaking up sitting time on cognitive functions. Abbreviations: BDNF – brain derived neurotrophic factor; DOPA – dihydroxy phenylalanine; PA- physical activity

The present public health approaches focus on strategies to improve moderate-vigorous physical activity in a large proportion of people (Fig. 1). Strategies to reduce and break up sitting time have only started to emerge, that have a potential to improve an individual’s cognitive function and brain health. The present mapping review will also shed light on the plausible direct and indirect hypothetical mechanisms associated with breaking up prolonged sitting on cognitive functions such as memory, attention, visuospatial skills and executive functions in sedentary adults.

### Excessive sitting and altered cognitive function

In modern computerized workplace settings, work productivity depends on the following cognitive components: skill acquisition, learning, attention, working memory, executive functions and decision-making [28]. There is some preliminary, though inconsistent, evidence on direct and indirect physiological mechanisms linking prolonged sitting with adverse cognitive outcomes [12, 13, 29]. Some of the proposed mechanisms include insufficient cerebral glucose utilization due to altered postprandial hyperglycemia [21], altered cortical hypoxemia due to compromised ventilatory volumes and peripheral vascular dysfunctions [12], poor arousal because of insufficient supply of Brain-Derived Neurotrophic Factor (BDNF) [30] and interacting hormones such as cortisol and dihydroxyphenyl alanine (DOPA) [13, 29]. Further, poor brain metabolism might increase reactive oxygen species and interleukins that could increase fatigue and reduce synaptic plasticity and memory [31]. The physiological mechanisms underpinning excessive sitting on cognitive functions are explained below.

#### How prolonged sitting could influence brain glucose metabolism?

**Fig. 2:**
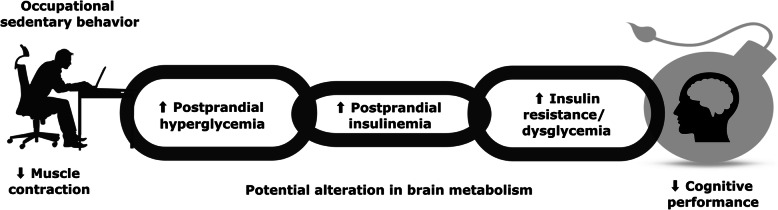
Possible effects of prolonged sitting on glucose metabolism in brain. Experimental trials have revealed a significant increase in postprandial hyperglycemia, insulinemia and insulin resistance among individuals sitting for extended periods. This may substantially reduce brain glucose metabolism and cognitive functions

Glucose is the primary fuel source for brain metabolism and function. Brain blood glucose transport and neural activity are significantly reduced after exposure to postprandial hyperglycemia [32]. Further, hyperglycemia and insulin resistance can be anticipated as causes for poor cognitive skills. Hyperglycemia and insulin resistance in individuals without clinically apparent diabetes, is found to be associated with reduced cognitive measures, with an atrophy of the hippocampus [33]. Contemporary evidence claims that prolonged uninterrupted sitting elevates postprandial hyperglycemia, proportional hyperinsulinemia and subsequent insulin resistance [34]. Nevertheless, the above metabolic derangements negatively affect brain glucose metabolism that would result in reduced cognitive performance (memory impairment) and cognitive decline [21] as depicted in Fig. 2.

#### How prolonged sitting might influence brain structure and activity?

**Fig. 3:**
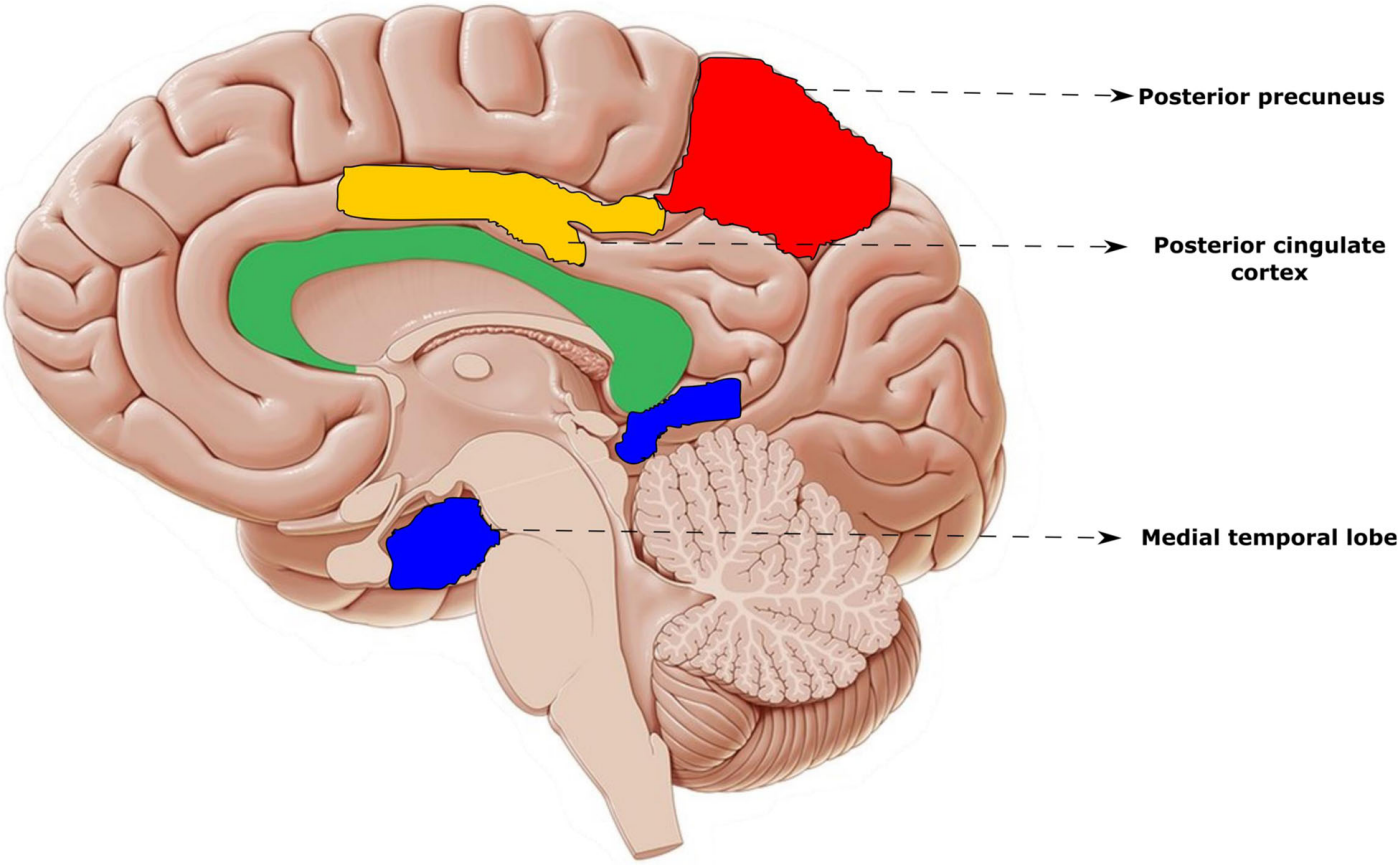
Cortical regions associated with attention and working memory related with sedentary behavior

Explicit episodic memory and attention is associated with medial temporal lobe (MTL) activity [35]. Moreover, decreased posterior cingulate and precuneus activity is found to be correlated with better episodic memory [35]. Given that the MTL, specifically the hippocampus, is one of the primary targets of physical activity effects in the rodent brain [36, 37] and age-related neurodegeneration (and Alzheimer disease) in humans [38, 39], prolonged sitting is speculated to reduce the MTL density and activity [40]. Figure 3. shows the cortical regions (medial temporal lobe, posterior cingulate cortex and posterior precuneus) associated with attention and working memory that might be influenced by sedentary behavior.

Sedentary behavior has been associated with poor posterior cingulate gyrus and medial temporal lobe (MTL) density and the future risk of neurodegenerative disorders [41]. In a fairly recent cross-sectional study, Siddharth et al. (2018) found an inverse correlation between sitting hours/day and MTL thickness (parahippocampal [*r* = − 0.45, *p* = 0.007], entorhinal [*r* = − 0.33, *p* = 0.05] and subiculum [*r* = − 0.36, *p* = .04] regions) which may negatively impact cognitive functions [40]. In a fairly recent randomized controlled trial (Inphact Treadmill study), Bergman et al. compared long term effects of treadmill desks in a group of 40 overweight and obese office workers to conventional education alone on magnetic resonance imaging of the brain volume for 13 months [42]. Longitudinal mediation analysis revealed non-significant reduction in hippocampal volume (− 33 mm^3^) and anterior cingulate cortex (− 0.02 mm) in control group compared to treadmill desk group at 13 months. Further hippocampal volume was positively correlated with the walking time [*β* = 1.448] and negatively correlated with total sitting time [*β* = − 0.462] [42]. Thus, excessive sitting may be detrimental to the medial temporal lobe, posterior precuneus and posterior cingulate cortex density which may be speculated due to reduced neuronal potentiation, cerebral blood flow and synaptic plasticity. This reduction in structural cortical density is postulated to the early cognitive decline and dementia risk [43].

#### How prolonged sitting could influence cerebral and peripheral vascular functions?

A decline in peripheral arterial function (predominantly endothelial shear and flow mediated dilation) is perceived to compromise the cardiovascular function and alter the cortical hemodynamics. It is speculated that excessive sitting may modify the anatomy of the lower limb arteries and form unprecedented changes to hemodynamics (e.g. increased venous pooling, stasis and blood viscosity), thereby compromising preload volume to heart [44]. Further, excessive sitting for > 6 h is found to be associated with reduced shear stress in the lower limb blood vessels which may alter endothelial integrity and consecutively result in endothelial dysfunction in the lower limbs [45]. Venous pooling, loss of endothelial integrity and viscous blood flow in the lower limbs may compromise the central hemodynamics and cortical circulation [46]. Recently, Paterson et al. (2020) systematically reviewed 17 studies that investigated the effects of prolonged sitting compared to interrupted sitting strategies (standing, walking or calisthenics) on peripheral vascular functions in adults [47]. Lower limb flow mediated dilation was found to be reduced by 2.12% (95% CI − 2.66 to − 1.59) during sitting bouts lasting more than 1 h [47]. Another speculated mechanism for cerebral hypoperfusion is hyperglycemia, that causes endothelial damage and reduce shear stress; however, based on the recent review such evidence exists only for animal models [21]. Thus, prolonged poor cerebral perfusion may reduce oxygen supply to the brain, and disrupt neuronal metabolism, and damage astrocytes and microglial cells leading to an impairment in learning and working memory [48].

#### How prolonged sitting could influence respiratory functions?

Compromised respiratory functions may hamper the optimal alveolar recruitment and available alveolar oxygen (P_A_O_2_) necessary for cortical oxygenation and, subsequently, affect cognitive function [49] following prolonged sitting. Prolonged sitting for more than an hour was found to increase slouched posture and forward leaning which are found to cause fatigue in rectus abdominis, internal oblique, transversus abdominis and iliocostalis muscles in office workers [50]. The thoracolumbar core muscles fatigue in turn may reduce lung volumes and capacities [51] resulting in reduced alveolar ventilation. The above speculation can be confirmed from the findings of a fairly recent systematic review by Katz et al. (2018) [52]. The authors found an average increase of 0.21 L in forced expiratory volume in 1 sec (FEV_1_) and forced vital capacity (FVC) with standing than sitting from a pooled analysis of 43 studies with only 26 studies involving healthy participants [52]. In a large stratified study involving 51,338 Canadian healthy adults and patients with lung disease, sitting time was negatively associated with FEV_1_ and FVC (β = − 0.32, CI: − 0.2, − 0.54) [53]. In addition, the sitting posture was found to reduce vital capacity, functional residual capacity and peak expiratory flow (PEF) compared to the standing posture in brass players [54]. In another study, prolonged sitting for 1 h in a chair with backrest compared to that without backrest is found to significantly reduce dynamic lung volumes (PEF = − 0.29 L/min; FEV1 = − 0.15 L; FVC = − 0.10 L) in 24 Korean adults [55]. Thus, reduction in dynamic lung volumes in sitting may reduce the alveolar recruitment and reduce the availability of continuous oxygen supply to the brain.

#### How prolonged sitting might affect hormonal function?

Hormones such as Brain Derived Neurotrophic Factor (BDNF), Dihydroxy Phenyl Alanine (DOPA) and Dihydroxy Phenyl Glycol (DHPG) are found to improve sympathetic activity resulting in higher frontal lobe functions [56]. In an experimental trial, Wennberg et al. (2016) demonstrated that increased fatigue is associated with a decrease in heart rate (*r* = − 0.60, *p* = 0.007) and plasma level of DOPA (*r* = − 0.59, *p* = 0.009) and an increased level of plasma dihydroxyphenyl glycol (DHPG; *r* = 0.73, *p* < 0.001) at first 4 h of uninterrupted sitting [13] in 19 sedentary desk-based Australian workers. The central fatigue due to increased cortisol levels seems to be negatively associated with cognitive function (primarily executive functions) [13]. In contrary, Sperlich et al. (2018) found no difference in plasma cortisol levels between prolonged sitting for 3 h and sitting interrupted with high intensity interval training for 6 min after 1 h of sitting in 12 young adults [30]. Hence, there are inconsistent findings to substantiate that prolonged sitting will affect salivary cortisol and stress hormones.

**Fig. 4:**
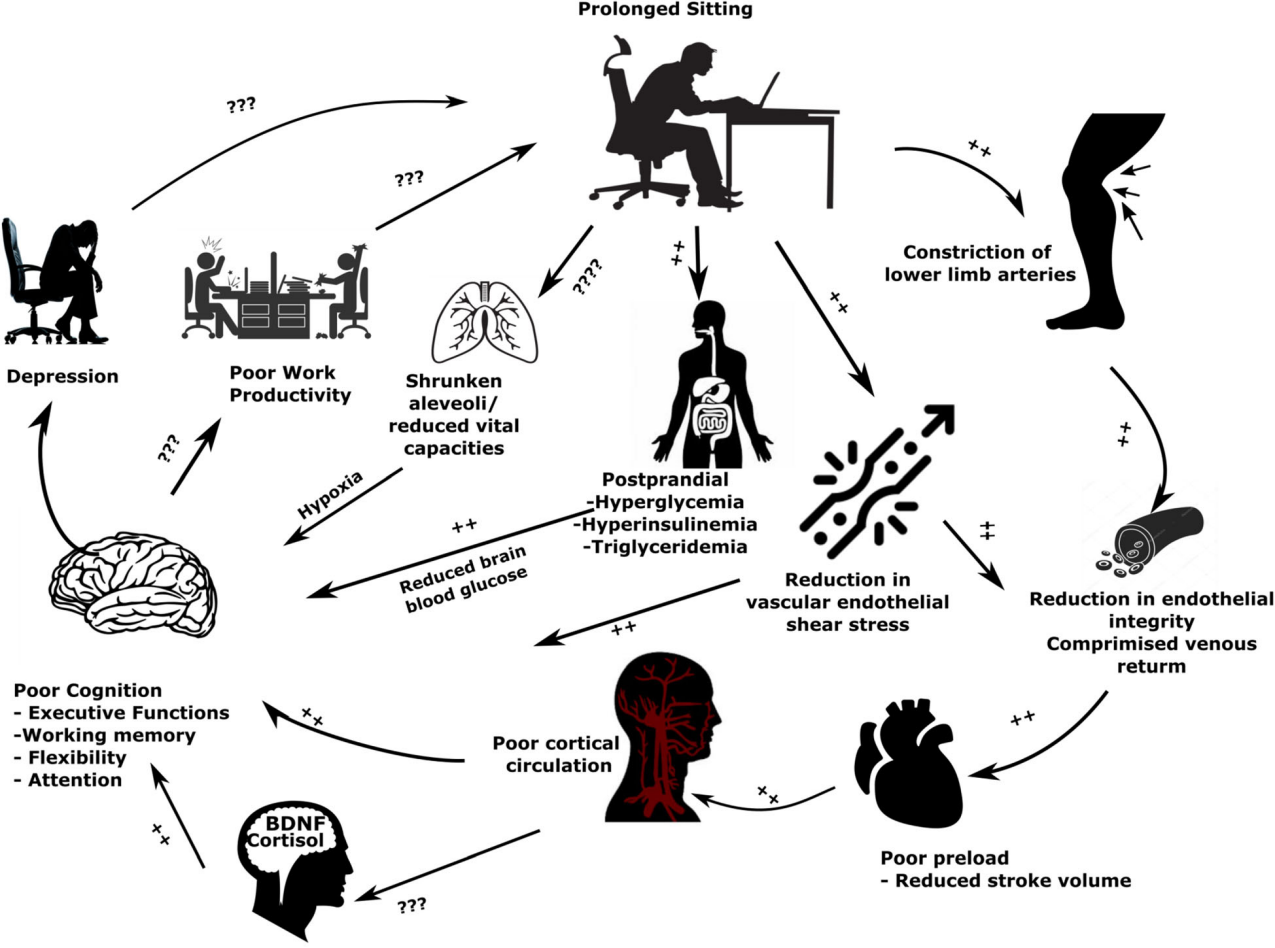
Prolonged sitting is viewed as a risk factor for poor cognitive functions because of aberrant physiological changes in the cardiovascular, pulmonary and metabolic systems. Poor cognitive functions might be associated with prolonged sitting during working hours, and negatively affect work productivity as a vicious cycle. Note: ++ denotes established evidence from experimental trials;??? represents a hypothetical link; BDNF – Brain derived neurotrophic factor

Based on the physiological mechanisms presented above, we hypothesize that prolonged sitting is likely to affect cognitive functions. Figure 4 demonstrates the hypothetical model of possible interactions of the physiological mechanisms linked to excessive sitting. Therefore, breaking up prolonged sitting is viewed as an imperative strategy for improving cognitive functions.

### Effects of breaking sitting on cognitive performance

Breaking up prolonged sitting with standing or low-to-moderate intensity exercise breaks is an effective intervention to induce changes within the physiological systems [57–59]. The proposed physiological mechanisms are interrelated, that might influence cognitive functions. We propose that there are direct and indirect effects of breaking up prolonged sitting on cognitive functions.

#### Direct effects of breaking up prolonged sitting on cognitive functions

Emerging evidence claims the direct influence of breaking up prolonged sitting seems conflicting. In an experimental trial, Schwartz et al. (2017) assessed the reaction time (Stroop test), working speed (text editing task) and attention (d2R test of attention) in 45 students (aged 25.4 ± 3.3 years) in two alternating postures (sitting and standing) [16]. The authors found no significant difference in cognitive functions between sitting and standing postures. Similarly, a recent crossover experimental trial by Vincent et al. (2018) investigated the effects of prolonged sitting and breaking up sitting periods in six males on three consecutive sleep restricted days [60]. The average reaction time on the psychomotor vigilance test and digital symbol substitution tests did not differ between prolonged sitting and sitting interrupted with breaks. However, another crossover trial by Christmas et al. (2019) compared attention, episodic memory and executive functions between the conditions of breaking up sitting (3 min of treadmill walk every 30 min for 5 h) and uninterrupted sitting (5 h) in 11 sedentary Qatari females [12]. The authors found quicker reaction times (~ 210 ms) in Stroop incongruent task test following breaking up sitting compared to uninterrupted sitting [12]. Moreover, Wheeler et al. (2019) administered three behavioral interventions: 1) uninterrupted (SIT); 2) moderate exercise for 1 h before uninterrupted sitting (SIT+Ex) and 3) combined exercise and scheduled breaks every 30 min (SIT+Break) in 67 Australian community dwelling adults and found a significant improvement in working memory and executive functions in SIT+Break and SIT+Ex than SIT groups [61].

#### Indirect but interlinked mechanisms of breaking up prolonged sitting on cognitive functions

**Fig. 5:**
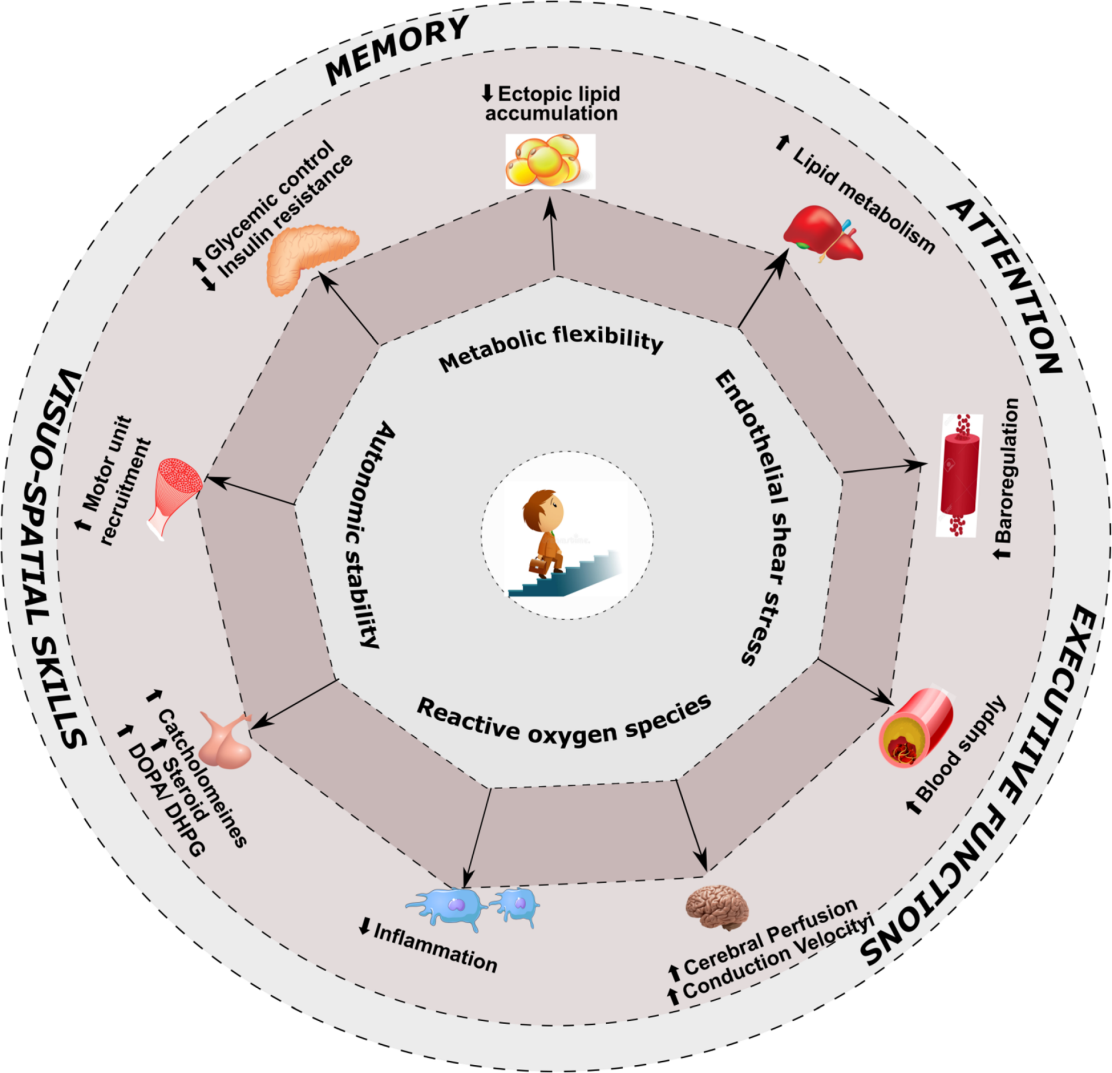
Physiological change wheel demonstrating the impact of interrupting sitting with physical activity at the workplace on plausible physiological systems and cognitive functions. DOPA – Dihydroxy phenylalanine; DHPG - Dihydroxy phenyl glycol, increase; decrease

Breaking up prolonged sitting seems to improve or regulate certain indirect physiological mechanisms that might influence cognition. The plausible physiological mechanisms are modulating: 1) cardiometabolic risk markers, 2) glucose metabolism, 3) adipose tissue metabolism, 4) energy expenditure and metabolic preference, 5) neuroendocrine functions, 6) muscular system and 7) central and peripheral vascular functions. Figure 5. depicts the direct and indirect effects of breaking up sitting on cognitive functions.

#### Effects of breaking prolonged sitting on cardiometabolic disease risk

Breaking up prolonged sitting may reduce postprandial hyperglycemia, insulin resistance and endothelial damage, thereby improving hemodynamics and cardiovascular health [18]. A recent meta-analysis by Hadgraft et al. (2020) of ≤33 studies revealed a significant change in both anthropometric measures (weight [− 0.6 kg], waist circumference [− 0.7 cm] and body fat percentages [− 0.3%]) and cardiometabolic risk factors (blood pressure [− 1.1 mmHg], plasma insulin [− 1.4 pM] and increased high-density lipoprotein cholesterol [+ 0.04 mM]) following interventions targeting sedentary behavior reduction with or without an increase in physical activity [18]. Another meta-analysis (of ≤24 studies) by Mulchandani et al. (2019) found that worksite based physical activity interventions (interrupting prolonged sitting) have significantly reduced body weight (− 2.61 kg), BMI (− 0.42 kg/m^2^) and waist circumference (− 1.92 cm); however, the analysis did not find a significant change in blood pressure, lipid profile and blood glucose levels [62]. The significant reduction in lipid profile and blood glucose in the review by Hadgraft et al. (2020) may be due to stringent search criteria, larger number of studies and sensitivity analysis. It could be hypothesized that breaking up sitting bouts may improve postprandial glucose metabolism, insulin resistance, lipid profile and other cardiometabolic risk biomarkers such as inflammatory markers. Though the reduction in cardiometabolic risk factors associated with the breaking sedentary behavior is perceived to regulate endothelial integrity, cortical perfusion and thus may improve cognitive functions [21], early experimental mechanistic studies failed to establish the relation between cardiometabolic risk factors, endothelial functions and cognitive functions [63, 64].

#### Effects of breaking prolonged sitting on cortical and peripheral glucose metabolism

Brain metabolism depends entirely on glucose as energy source with 100–150 mg/day which constitutes nearly 20% of body glucose stores, although the brain accounts for only 2% of the body weight [65]. Glucose Transporter 1 (GLUT1) in the luminal and abluminal surfaces of blood brain barrier are responsible for active transport of glucose molecules across the tight blood brain barrier and cortical blood glucose utilization by the parenchymal cells which is facilitated by the concentration gradient [65]. Sedentary behavior including excessive sitting may result in reduced concentration gradient, and plasma hyperglycemia can alter the permeability of blood brain barrier and reduce the sensitivity of GLUT1 transporters which can lead to brain hypoglycemia [21]. Hence, breaking sitting can be viewed as an imperative solution to improve GLUT1 sensitization for improving glucose transport across blood-brain barrier, in turn central utilization of glucose and speculated to improve cognitive functions. Increased muscle contraction mediated glucose metabolism with acute standing [66] or walking bouts [67] has been perceived to improve brain glucose metabolism and cognitive functions [21, 61].

Prolonged sedentary bouts may be associated with cerebral hypoperfusion and this in turn is associated with neuroglycopenia, neural astrocyte and microglial damage resulting in poor cognitive functions [68]. Intermittent standing (e.g. every 20 min) is found to reduce postprandial glucose and insulin through upregulation of glucose transporters (GLUT1 and Glucose Transporter 2 in brain; Glucose Transporter 4 in muscle) [20, 21]. A recent systematic review by Loh et al. (2020) found a significant reduction in glucose (effect size, *d =* − 0.56; 95 CI -0.70, − 0.30) and insulin (*d = −* 0.56; 95% CI − 0.74, − 0.38) after physical activity at 40–70% of VO2max or self-selected walk breaks every 30 min to 1 h for 2 min to 30 min over a 7–9 h typical working hours [19]. Even so, another systematic review by Sauders et al. (2018) found that breaking up prolonged sitting has reduced postprandial glucose (d = − 0.36) and insulin levels (d = − 0.37), but not plasma triglyceride levels (d = 0.06) [69]. Therefore, we postulate breaking-up prolonged sitting can facilitate glucose metabolism and may provide sufficient glucose to the brain and subsequently improve cognitive functions [70]. Thus, improved cortical glucose utilization and peripheral glucose regulation with the microbreaks may be viewed as imperative mechanisms for cognitive functions improvement.

#### Effects of breaking prolonged sitting on adipose tissue and inflammatory markers

In a counterbalanced crossover trial, perivascular adipose tissue inflammation was hypothesized to be linked with the incidence of vascular diseases [71]. The study found a significant reduction in adipose tissue mRNA expression for several inflammatory genes including but not limited to interleukin 6, leptin, adiponectin, pyruvate dehydrogenase kinase and insulin receptor [71]. In a randomized controlled trial, Grace et al. (2019) demonstrated the metabolic effects of breaking up prolonged sitting on the adipose tissue transcriptional changes [72]. Breaking up prolonged sitting has been found to be positively associated with the regulation of lipid metabolism and inflammatory pathways [72], increased insulin signaling and adipocyte cell cycle regulation [18]. The above physiological changes may be associated with a reduction in inflammatory markers such as interleukin-6 (IL-6) and free radicals. Therefore, breaking up sitting could significantly reduce the proinflammatory cytokines such as tumor necrosis factor-α (TNF-α) and IL-6 which in turn may reduce macrophages formation in the subendothelial space, forming foam cells, and resulting atherogenesis and cerebral vascular functions [73]. However, further studies based on robust randomized clinical trials are warranted to evaluate the long-term effects of breaking up sitting with appropriate interventions on inflammation or vascular functions [18].

#### Effects of breaking prolonged sitting on energy expenditure

Brain metabolism accounts for 20% of total resting metabolism of the body [21]. Dynamic changes in brain glucose metabolism are of paramount importance for neuronal activation, neural plasticity and cognitive functions [21]. During thought process, an increase in neuronal metabolism depends on the availability of substrates and overall glucose metabolism of the body. Also, cerebral mitochondrial functioning depends on the cerebral glucose and neurometabolic-vascular coupling [48].

**Fig. 6:**
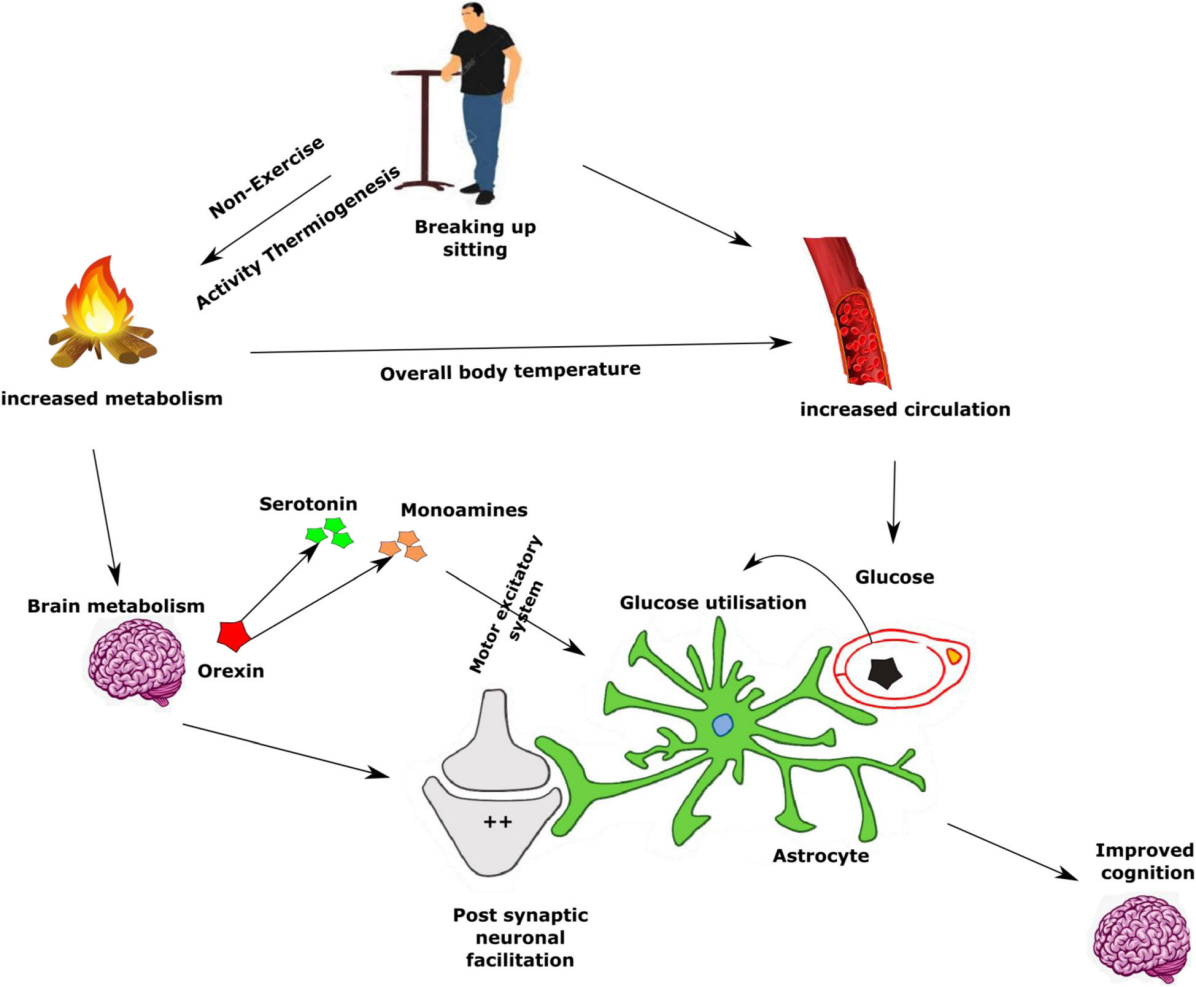
The energy expenditure resulting from breaking up prolonged sitting might increase brain metabolism by increasing orexins (neuropeptides) and improving cortical circulation. The orexins in turn might stimulate motor excitatory systems (norepinephrine, serotonins) and arousal stimulants (monoamines, acetylcholine)

Oxidative stress is linked with over nutrition, obesity and reduced energy expenditure [74]. Reduced energy expenditure is associated with changes in cellular oxidative state (mitochondrial enzymes and glycolytic pathway changes), especially an increase in reactive oxygen species or hypoxia, that induce cellular stress and damage [74]. Furthermore, orexin, an excitatory hypothalamic neuropeptide, which increases with voluntary physical activity, is found to increase brain metabolism, facilitate serotonin secretion from brainstem, and improve daytime wakefulness [75]. Orexin is argued to be reduced in obese and elderly populations and, claimed to be associated with poor cognitive functions which is depicted in Fig. 6.

Reduced energy expenditure is argued to be negatively associated with cognitive functions [76]. Individuals with obesity/overweight have been found to have worse cognitive outcomes compared to their lean counterparts, mainly those related to executive functions [77]. Middleton et al. (2013) investigated the relationship between cognitive functions (based on mini mental state examination) and energy expenditure (estimated by double labelled water technique) in 323 community dwelling older adults through a prospective cohort study [78]. The older adults with a high energy expenditure had lower odds of cognitive impairment incidence (odds ratio [OR]: 0.09; 95% CI 0.01–0.79) compared to those with a low energy expenditure. In another (cross-sectional) study of 123 community dwelling, non-demented adults, energy expenditure independently accounted for a 2% variance in verbal learning and the delayed verbal recall on the Rey Auditory Verbal Learning Test [76]. Thus increased energy expenditure and improved glucose metabolism could be speculated to improve the executive functions such as response inhibition and information processing speed among adults [58] but the convincing evidence has still yet to be investigated.

#### Effects of breaking sitting on the neuroendocrine functions

Though facilitation of the sympathetic nervous system and neural hormones such as epinephrine and norepinephrine during postural change have been investigated for decades [79], its role in arousal and cognitive functions have not been substantiated. Chronic fatigue following excessive sitting may be associated with impaired autonomic nervous system functions [13]. In a crossover trial, intermittent walks have been found to improve the fatigue (visual analog scale – VAS-F) and cognitive functions – episodic memory (face-name association test), inhibition (Eriksen flanker and Stroop tests) and executive function updating (n-back and letter memory test) as compared with uninterrupted sitting in a 7-h experimental condition [13]. The probable causative mechanism through which breaking up prolonged sitting strategies reduce the fatigue is through autonomic nervous system regulation. Increased sympathetic nervous system facilitation, increased release of adrenalin, norepinephrine and increased metabolism and continuous brain glucose supply are hypothesized to reduce central fatigue and improve cognition [13].

Breaking up prolonged sitting or exercise may increase the dopamine, catecholamine levels in the brain [59]. Consequently, the brain catecholamines might increase arousal by activating the reticular formation [80]. Further, the interaction between hypothalamic-pituitary-adrenal cortex (HPA) axis hormones and BDNF is found to be significantly associated with cognitive functions [80]. Though the brain is the major source of BDNF, the circulatory enzymes secreted by muscles such as irisin, cathepsin B, or liver-derived β-hydroxybutyrate during exercise/physical activity are also postulated to positively influence BDNF which is found to be associated with learning and memory related cognitive functions [61].

In a pilot crossover trial by Wennberg et al. (2016), the participants underwent either uninterrupted sitting for 7 h or sitting interrupted with a 3-min walk every 30 min for 2 days with a wash-out period of 6 days in-between [13]. The authors found a significant reduction in fatigue levels and improvement in composite cognitive scores from the Face-Name Association, Eriksen Flanker test, Stroop color test, N-back and letter memory test at 4 h and 7 h of sitting. Also, fatigue scores over time correlated with a decrease in heart rate and plasma DOPA and an increase in plasma DHPG [13]. In the three arm cross over trial by Wheeler et al. (2020), 8 h serum BDNF was found significantly higher in two intervention days among 65 Australian sedentary obese office workers [61]. Their participants received morning exercise with breaks every 30 min (EX+BR) or without breaks for the next 6.5 h (EX+SIT) [171 (− 449 to + 791) ng/mL·hour] compared to uninterrupted sitting (SIT) day for 8 h [− 227 (− 851 to + 396) ng/mL·hour] [61]. However, the authors did not find significant difference between the intervention groups either EX+SIT or EX+break [61]. Despite of the growing anecdotal evidence, empirical evidence seems to remain equivocal regarding the beneficial effects of breaking up prolonged sitting on neuroendocrine functions.

#### Effects of breaking up prolonged sitting on muscular system

Disrupting monotonous muscle activity with scheduled breaks is postulated to regulate isokinetic peak torque and power. These physiological changes are considered to reverse reduced motor unit potentiation and neuromuscular fatigue associated with prolonged sitting (Fig. 5) [14]. However, the current empirical evidence does not favor the (neuromuscular) effects of breaking up sitting, especially, on skeletal muscle twitch amplitude and fatigue accrued during the typical work hours [81].

#### Effects of breaking prolonged sitting on cerebral and peripheral vascular dynamics

**Fig. 7:**
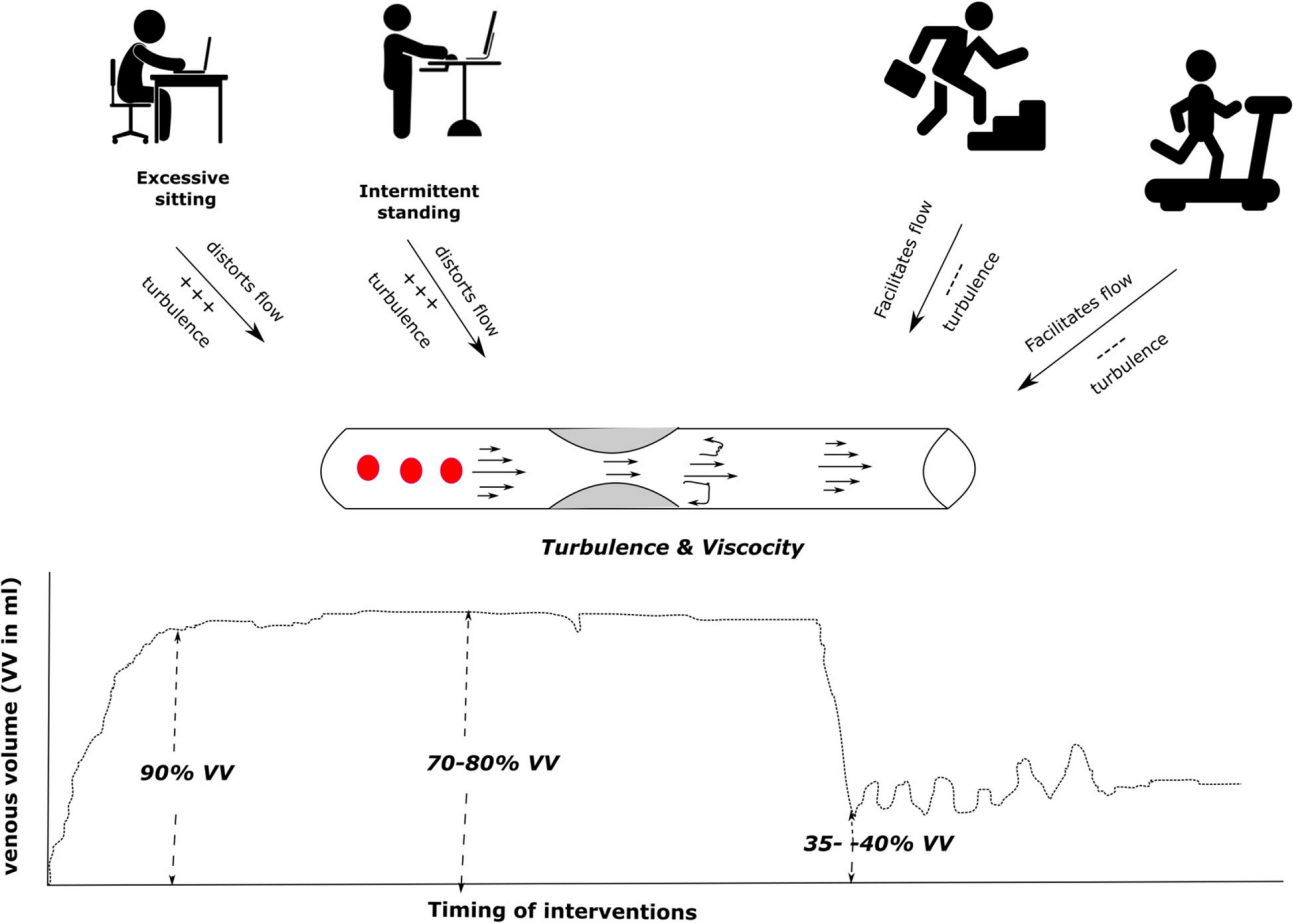
Breaking sitting with low-to-moderate intensity physical activity is postulated to improve the vascular functions by improving the venous return. This improvement may be due to streamlining of the blood flow, flow mediated dilation and endothelial shear stress augmenting venous return and improved cortical perfusion

Breaking up sitting bouts with short bouts of physical activity is postulated to improve endothelial functions, regulating peripheral, cerebral blood flow, improving venous return (Fig. 7) and viewed as a prophylactic strategy to mitigate impaired cognitive functions associated with prolonged sitting [57]. In a recent systematic review, Paterson et al. (2020) analyzed the effects of interrupted bouts with aerobics, resistance exercises or standing on flow mediated dilation of brachial, femoral and posterior tibial arteries from 6 studies [47]. The review found significantly higher flow mediated dilation (1.91%; 95% CI 0.40–3.42%) during interrupted sitting bouts compared to uninterrupted sitting [47]. However, the dose response relationship of the interventions targeting reducing sitting or improving physical activity on the cerebral flow velocity remains unclear.

In a crossover trial, 15 middle-aged adults underwent three interventions: uninterrupted sitting for 4 h, sitting interrupted with a 2-min walk every 30 min and sitting with a 8-min walk every 2 h [57]. The study found a significant decline of cerebral flow velocity in uninterrupted sitting (− 3.2 ± 1.2 cm/s) compared to 2-min walk breaks (0.6 ± 1.5 cm/s) but not 8-min walk breaks (− 1.2 ± 1.0 cm/s). In contrary to earlier studies, Maasakkers et al. (2020) failed to find a significant difference in cerebral flow velocity or cerebral vasomotor functions between uninterrupted (3 h) and interrupted sitting bouts (2 min walk break every 30 min for 3 h) combined with or without mental tasks in 22 elderly adults (78 years) [63]. The aforementioned inconsistent findings regarding the effects of interrupted sitting bouts on the cerebral flow velocity and perfusion makes it difficult to extrapolate the effects of such interventions on cognitive functions.

Few promising controlled trials have succeeded in observing a change in peripheral blood vessels shear stress and flow mediated dilation percentage after an acute exposure of breaking up sitting with different doses of physical activity (low intensity walks to moderate intensity exercises) [82, 83]. Thosar et al. (2015) interrupted 3 h of sitting with active breaks which prevented a decline in superficial femoral artery dilation (0.24–1.74% from the baseline) with no reduction in shear rates in 12 non-obese men [82]. In addition, Carter et al. (2019) in an experimental study investigated the effects of two different break strategies (2-min walking breaks every 30 min of sitting and 8-min walking breaks every 2 h) on superficial femoral artery endothelial function in 15 healthy desk-based office workers. They found an increase in SFA blood flow (by 0.45 ± 17.7 mL·min) after 8-min breaks compared to 2-min breaks [83]. The observed increase in blood flow and arterial dilation is perceived as a necessary mechanism for reducing the risk of atherosclerosis and future cardiovascular diseases [44]. This improved vasodilation is probably due to improved nitric oxide (endothelium-derived relaxing factor), prostaglandin and increased venous return which in turn may improve the cortical perfusion [84]. In a recent study, Carter et al. (2020) assessed the effects of an e-health intervention on vascular function, amongst other outcomes, in 14 heathy office workers for 8 weeks. In spite of perceived interruption to the routine workflow, vascular functions improved (d = 0.88) and total daily sitting time (d = 0.92) decreased with the scheduled breaks delivered through the e-health intervention [85].

### Summary of the hypothesis

Prolonged (excessive) sitting at workplaces leads to vascular and cardiometabolic changes that predispose to both peripheral and central vascular inflammation and poor cortical perfusion might lead to poor cognitive function. With a myriad of ill effects associated with excessive sitting, breaking or reducing sitting needs to be visualized as a necessary measure to mitigate cardiovascular/metabolic risks and poor cognitive function.

Executive functions are crucial for improving work productivity. Hence, breaking up prolonged sitting can indirectly influence work productivity in sedentary office workers by various physiological mechanisms (Fig. 4.) for which the evidence is still emerging.

Occupational interventions, amongst others, such as using height-adjustable desks, participating in standing/walking meetings, environmental restructuring (e.g. stairwells), restricting elevators usage, active commuting to work and physical activity counselling are some attempts to reduce occupation-related sedentary behavior [86–89]. Contemporary evidence claims workplace digital interventions such as e-health may influence cardiometabolic disease risk especially mean arterial pressure (MAP) [90, 91]. A 12-month e-health intervention was found to reduce MAP (3.6–4.0 mmHg) significantly compared to baseline. A plethora of evidence exists to emphasize the effects of unimodal or multimodal behavioral interventions to improve sedentary behavior and minimize excessive sitting bouts during working hours [87, 89]. Despite such growing evidence on positive effects of reducing prolonged sitting on health outcomes, sitting behavior seems to be increasingly prevalent globally [92]. Advocating 1 h of moderate-to-vigorous physical activity at gyms for office workers (sitting more than 6 h/day) seems less feasible. Rather frequent light intensity activities during typical work hours could be advocated for mitigating the detrimental effects associated with prolonged sitting.

### Strengths and limitations of the study

The present mapping review presents concepts and hypotheses based on theories stemming from the available evidence underpinning the physiological effects of the breaking up sitting on the cognitive performance. The results may inform the stakeholders about framing research questions and conducting primary studies to address the evidence gap in this area and also identify the need to address cognitive performance in the existing physical activity guidelines. Employing a pragmatic search and a critical interpretive synthesis, we have searched, identified and included reviews and primary studies based on their relevance to our research question. Gray literature and non-English studies were not included. Even so, unlike systematic reviews of effectiveness of interventions, we expect that adding a few more studies is less likely to change the concepts and physiological mechanisms mapped. Moreover, we did not assess the methodological quality (risk of bias) of the included studies as it’s not advocated for mapping reviews. These methodological considerations should be noted while interpreting the findings of the mapping review.

### Research implications

Though the hypothesis seems to be thriving well, based on this mapping review, for investigating the effects of breaking up prolonged sitting on the cognitive functions, the evidence underpinning the hypothesized physiological mechanisms is at its infancy. Still there are several questions that remain unanswered: 1) dose response (frequency, intensity and duration) of scheduled breaks on the cognitive functions is still unclear; 2) the molecular mechanisms by which breaking up prolonged sitting with or without exercise/physical activity improve cognitive function remains ambiguous; 3) the age and gender differences in responses to breaking up prolonged sitting needs further investigation.

## Conclusion

Breaking up prolonged sitting by intermittent standing or low to moderate physical activity may be an imperative measure for improving cognitive function by mitigating the ill-effects (aberrant vascular and hormonal changes) associated with the excessive sitting. Positive effects of breaking up sitting on postprandial hyperglycemia, insulin resistance, inflammatory markers (especially IL-6), hormonal (cortisol, DOPA and DHPG) regulation, and cortical and peripheral arterial blood flow are viewed as mechanistic links to negate the cognitive decline associated with prolonged sitting.

It is evident from our mapping review that there is a paucity of research substantiating the physiological mechanisms underpinning the effects of breaking up prolonged sitting on cognitive functions in sedentary adults. Further primary studies and subsequent systematic reviews are warranted to ascertain the empirical evidence for the physiological (hypothetical) mechanisms postulated in the present review.

## Supplementary Information

**Additional file.1:** Sample search strategy used in Ovid Medline.

**Additional file.2:** Flowchart showing the iterative phases developed and screening process for the present mapping review.

## Data Availability

Not Applicable.

## Abbreviations

CBF: Cerebral blood flow
BDNF: Brain-derived neurotrophic factor
DOPA: Dihydroxyphenyl alanine
MTL: Medial temporal lobe
P_A_O_2_: Partial pressure of alveolar oxygen
FEV_1_: Forced expiratory volume in one second
FVC: Forced vital capacity
PEF: Peak expiratory flow
DHPG: Dihydroxy phenyl glycol
GLUT1: Glucose transporter 1
IL-6: Interleukin-6
TNF-α: Tumor necrosis factor-α
HPA: Hypothalamic-pituitary-adrenal cortex
MAP: Mean arterial pressure
