# Supplementary material for: Does breaking up prolonged sitting improve cognitive functions in sedentary adults? A mapping review and hypothesis formulation on the potential physiological mechanisms
Source: BMC Musculoskelet Disord. 2021 Mar 12;22:274. doi: 10.1186/s12891-021-04136-5 (PMC7955618; doi:10.1186/s12891-021-04136-5)
Supplement: Supplementary file 1 — Additional file.1: Sample search strategy used in Ovid Medline. [file 12891_2021_4136_MOESM1_ESM.docx]

**Additional file:1. Sample search strategy administered in Ovid Medline for the present mapping review**

Database: Ovid MEDLINE(R) ALL <1946 to December 12, 2021>

Search Strategy:

--------------------------------------------------------------------------------

1 ((sedentar* or sitting) adj2 (behavio?r or time)).mp. [mp=title, abstract, original title, name of substance word, subject heading word, floating sub-heading word, keyword heading word, organism supplementary concept word, protocol supplementary concept word, rare disease supplementary concept word, unique identifier, synonyms] (15160)

2 ((Prolong* or uninterrupt) adj2 (sitting or sedentar*)).mp. [mp=title, abstract, original title, name of substance word, subject heading word, floating sub-heading word, keyword heading word, organism supplementary concept word, protocol supplementary concept word, rare disease supplementary concept word, unique identifier, synonyms] (1045)

3 ((workplace or office) adj2 (sitting or sedentar*)).mp. [mp=title, abstract, original title, name of substance word, subject heading word, floating sub-heading word, keyword heading word, organism supplementary concept word, protocol supplementary concept word, rare disease supplementary concept word, unique identifier, synonyms] (262)

4 ("movement break" or microbreak).mp. [mp=title, abstract, original title, name of substance word, subject heading word, floating sub-heading word, keyword heading word, organism supplementary concept word, protocol supplementary concept word, rare disease supplementary concept word, unique identifier, synonyms] (17)

5 ((breaking or interrupt* or disrupt*) adj2 (sedentar* or sitting)).mp. [mp=title, abstract, original title, name of substance word, subject heading word, floating sub-heading word, keyword heading word, organism supplementary concept word, protocol supplementary concept word, rare disease supplementary concept word, unique identifier, synonyms] (264)

6 (Cognition or memory).mp. [mp=title, abstract, original title, name of substance word, subject heading word, floating sub-heading word, keyword heading word, organism supplementary concept word, protocol supplementary concept word, rare disease supplementary concept word, unique identifier, synonyms] (448389)

7 (Cognitive adj2 (performance or function or decline)).mp. [mp=title, abstract, original title, name of substance word, subject heading word, floating sub-heading word, keyword heading word, organism supplementary concept word, protocol supplementary concept word, rare disease supplementary concept word, unique identifier, synonyms] (76562)

8 (attention or memory or focus).mp. [mp=title, abstract, original title, name of substance word, subject heading word, floating sub-heading word, keyword heading word, organism supplementary concept word, protocol supplementary concept word, rare disease supplementary concept word, unique identifier, synonyms] (1158187)

9 ("reaction times" or accuracy).mp. [mp=title, abstract, original title, name of substance word, subject heading word, floating sub-heading word, keyword heading word, organism supplementary concept word, protocol supplementary concept word, rare disease supplementary concept word, unique identifier, synonyms] (439478)

10 (cognitive adj2 (flexibility or inhibit*)).mp. [mp=title, abstract, original title, name of substance word, subject heading word, floating sub-heading word, keyword heading word, organism supplementary concept word, protocol supplementary concept word, rare disease supplementary concept word, unique identifier, synonyms] (4467)

11 6 or 7 or 8 or 9 or 10 (1719788)

12 1 or 2 or 3 or 4 or 5 (15714)

13 11 and 12 (1722)
