# Supplementary material for: Does breaking up prolonged sitting improve cognitive functions in sedentary adults? A mapping review and hypothesis formulation on the potential physiological mechanisms
Source: BMC Musculoskelet Disord. 2021 Mar 12;22:274. doi: 10.1186/s12891-021-04136-5 (PMC7955618; doi:10.1186/s12891-021-04136-5)
Supplement: Supplementary file 2 — Additional file.2: Flowchart showing the iterative phases developed and screening process for the present mapping review. [file 12891_2021_4136_MOESM2_ESM.docx]

**Additional file:2.**

Phase:1. Exploratory Reading

- A pilot random search was conducted and

four studies that investigated the sedentary effects on cognitive performance were identified

- Potential search terms were defined.

Phase:2. Databases search

- Four databases (Ovid, PubMed, CINAHL, Embase) were searched from their inception to December 12, 2020 using database-specific search terms
- Relevant articles that explored the physiological mechanisms of sedentary behavior and its interruptions which can be mapped further with the other factors influencing cognitive performance (n=18)
- Along with the primary articles, additional supporting evidence (n =34) which the authors deemed necessary for adequate mapping of physiological mechanisms underpinning the sedentary behavior on cognitive functions were included

Phase:4. Mapping the results

- A large number of citations were retrieved (n =1724) after initial search and duplicates removal;
- Studies investigating/exploring the physiological mechanisms underpinning the sedentary behavior and cognition were included for mapping (n= 52)

Phase:3. Screening of relevant articles

**Flowchart showing the iterative phases developed and included studies for the present mapping review**
